# Clarification of the molecular mechanisms underlying glyphosate-induced major depressive disorder: a network toxicology approach

**DOI:** 10.1186/s12991-024-00491-4

**Published:** 2024-01-31

**Authors:** Jianan Li, Haoran Bi

**Affiliations:** 1 Department of Occupational and Environmental Health, College of Public Health, Xuzhou Medical University, 209 Tongshan Road, Yun Long District, Xuzhou, 221000 China; 2 Department of Biostatistics, College of Public Health, Xuzhou Medical University, 209 Tongshan Road, Yun Long District, Xuzhou, 221000 China

**Keywords:** Major depressive disorder, Depression, Glyphosate, Exposure, Environmental factors

## Abstract

Major depressive disorder (MDD) is predicted to become the second most common cause of disability in the near future. Exposure to glyphosate (Gly)-based herbicides has been linked to the onset of MDD. However, the underlying mechanisms remain unclear. The aim of this study was to investigate the potential molecular mechanisms of MDD induced by Gly using network toxicology approach. The MDD dataset GSE76826 from the Gene Expression Omnibus database was referenced to identify differentially expressed genes (DEGs) in peripheral blood leukocytes of MDD patients and controls. The potential intersection targets of Gly-induced MDD were screened by network toxicology. The intersection targets were used for Gene Ontology (GO) and Kyoto Encyclopedia of Genes and Genomes (KEGG) enrichment analysis and to construct protein–protein interaction networks. The binding potentials of hub targets with Gly were validated by molecular docking. In total, 1216 DEGs associated with Gly-induced MDD were identified. Subsequent network pharmacology further refined the search to 43 targets. GO and KEGG enrichment analyses revealed multiple signaling pathways involved in GLY-induced MDD. Six potential core targets (CD40, FOXO3, FOS, IL6, TP53, and VEGFA) were identified. Finally, molecular docking demonstrated that Gly exhibited strong binding affinity to the core targets. The results of this study identified potential molecular mechanisms underlying Gly induced MDD and provided new insights for prevention and treatment.

**Supplementary Information:**

The online version contains supplementary material available at 10.1186/s12991-024-00491-4.

## Introduction

Major depressive disorder (MDD), commonly referred to as depression, has a significant impact on mental health, affecting approximately 3% of the global population, equating to approximately 216 million individuals [24]. Notably, individuals with depression may experience suicidal tendencies, underscoring the importance of early identification and prompt treatment of this debilitating condition [32]. MDD is anticipated to become the second most common cause of disability, particularly among the elderly population, by the year 2030 [14]. MDD is a complex disorder influenced by biological, genetic, and environmental factors [4].

Glyphosate (Gly) is a widely used active ingredient in herbicides, leading to widespread scrutiny of the potential toxicity of Gly-based formulations [22]. Worldwide, approximately 8.6 billion kilograms of Gly are applied to more than 350 million hectares annually [6]. Both in vivo and in vitro investigations have demonstrated toxic effects of Gly on both embryonic development and neurological systems [21]. Recent epidemiological investigations have demonstrated an association between Gly exposure and the incidence of MDD [11]. In particular, mice exposed to a low dose of Gly-based herbicide over a subchronic period exhibited behaviors resembling depression and anxiety [9]. In consideration of the wide use of Gly, potential harm to human health must be investigated, especially the potential molecular mechanisms underlying Gly-induced depression.

**Fig. 1:**
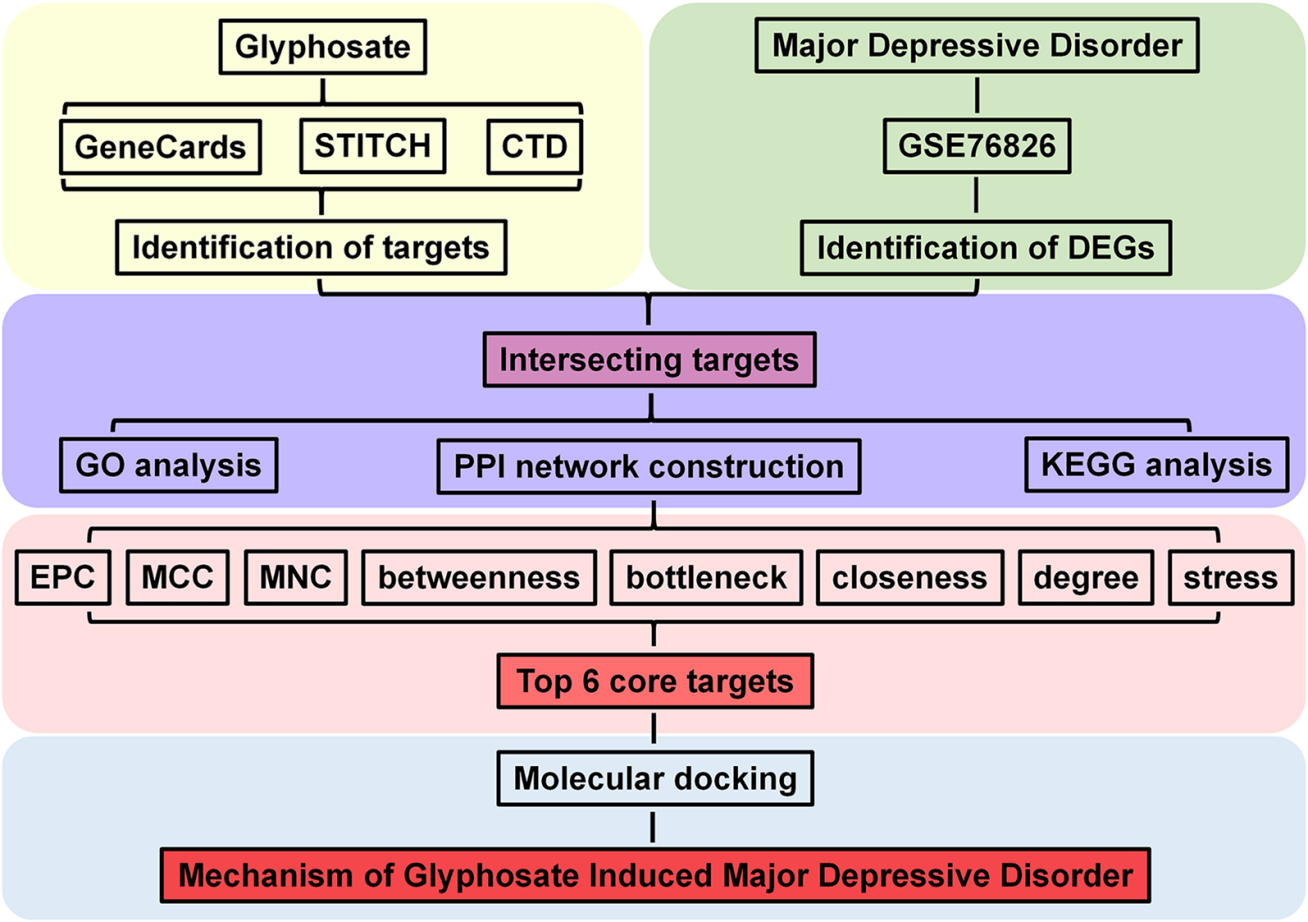
Study design and workflow

Therefore, the aim of the present study was to identify differentially expressed genes (DEGs) in peripheral blood leukocytes from MDD patients and controls. Furthermore, network toxicology and molecular docking strategies were used to screen potential targets of Gly exposure linked to the development of MDD. The results of this study indicate that exposure to Gly may be associated with the development of MDD and highlight the need for further investigation to elucidate the molecular mechanisms underlying this association. The study design and workflow are presented in Fig. 1.

## Materials and methods

### Genetic microarray data sources

The GSE76826 dataset was obtained from the Gene Expression Omnibus database (http://www.ncbi.nlm.nih.gov/). The study focused on nine older outpatients and inpatients (age ≥ 50 years) with MDD according to Diagnostic and Statistical Manual of Mental Disorders, 4th ed (DSM-IV) criteria. The severity of depression was assessed using the Hamilton Depression rating scale (SIGH-D) through structured interviews. In addition, 12 healthy individuals were included for comparison. Blood samples were collected, and RNA was extracted from peripheral blood leukocytes using the QIAmp RNA Blood Mini Kit. Microarray data were obtained based on the GPL17077 platform and conducted using the SurePrint G3 Human GE v2 8 × 60K Microarray (Agilent Technologies, Inc., Santa Clara, CA, USA). This study used publicly available gene data for analysis, and was therefore exempted from ethical approval.

### Identification of DEGs

The original data were recorded in the MINiML file format. The “normalize.quantiles” function from the “preprocessCore” package of the R software (version 3.4.1; https://www.r-project.org/) was used to standardize the microarray data. The normalized data platform provided annotation information, which was utilized to convert probes into gene symbols. All probes that matched multiple genes were eliminated from the datasets. The final expression value was calculated from the average expression value of genes that were identified by several probes. The criteria for MDD-related DEGs were |log2 fold change| ≥ 0.585 and a false discovery rate (FDR) < 0.05. Considering multiple testing, the Benjamini–Hochberg (BH) method was used in this study to adjust the *p* value to control for FDR. A difference was considered statistically significant if the BH-adjusted *p* value was less than 0.05.

### Network toxicology analysis

The GeneCards database of human genes (http://www.genecards.org/), STITCH database of known and predicted interactions between chemicals and proteins (http://stitch.embl.de/), and the Comparative Toxicogenomics Database (CTD, http://ctdbase.org/) were searched to identify potential targets of Gly-induced MDD. The two-dimensional structures of Gly were obtained from the PubChem database (https://pubchem.ncbi.nlm.nih.gov/). The CTD screening conditions were set to default parameters with the target species set to *Homo sapiens*. Similarly, the target species for searching of the STITCH database was set to *H. sapiens* and the minimum required interaction score was set to 0.4. The Draw Venn Diagram tool (http://bioinformatics.psb.ugent.be/webtools/Venn/) was used to identify the intersecting targets of Gly that may contribute to MDD. A protein–protein interaction (PPI) network was constructed of the intersecting targets using the STRING database (https://string-db.org/), with a minimum required interaction score of 0.15 for the species *H. sapiens*, and visualized using Cytoscape 3.6.1 (https://cytoscape.org/), an open source software platform for visualizing complex networks.

### Gene-set enrichment analysis

Gene-set enrichment analysis was performed using the Metascape gene annotation and analysis resource (https://metascape.org/gp/index.html) with reference to the Gene Ontology (GO) knowledgebase (http://geneontology.org/) and the Kyoto Encyclopedia of Genes and Genomes (KEGG; https://www.genome.jp/kegg/). The threshold of statistical significance was set to *p* < 0.05. The top 10 GO terms for biological process (BP), cellular component (CC), and molecular function (MF), in addition to the top 10 KEGG pathways were selected for further investigation.

### Hub targets screening

The cytoHubba plugin (https://apps.cytoscape.org/apps/cytohubba) was used to identify important nodes/hubs based on eight topological algorithms [betweenness, bottleneck, closeness, degree, edge percolated component (EPC), maximal clique centrality (MCC), maximum neighborhood component (MNC), and stress]. Then, the Draw Venn Diagram tool was employed to identify the top 10 overlapping (hub) targets.

### Molecular docking

To conduct molecular docking of Gly to the hub targets, the three-dimensional structure of Gly was retrieved from the PubChem database and converted into the Mol2 format using UCSF Chimera software version 1.16. In addition, the three-dimensional structures of the core targets were retrieved from the Worldwide Protein Data Bank (https://www.wwpdb.org/). Hydrogenation, dehydration, and ligand removal of the core targets were assessed with UCSF Chimera software (https://www.cgl.ucsf.edu/chimera/). The SwissDock online platform (http://www.swissdock.ch/) was utilized to perform molecular docking and to assess the binding activity of Gly and the hub targets. The results of the docking process were visualized using the UCSF Chimera software.

## Results

### DEG screening and network toxicological analysis

**Fig. 2:**
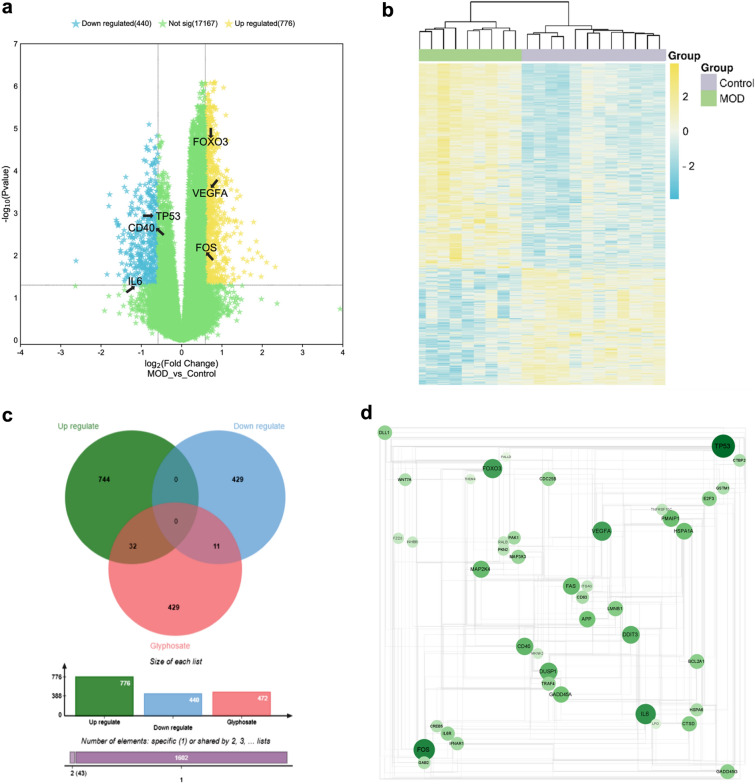
DEG screening and network toxicological analysis. **a** Volcano plot of DEGs between MDD patients and control. The hub targets were marked with black arrows. **b** Heatmap of DEGs between MDD patients and control. **c** The Venn map of Gly-related targets and MDD-related targets. **d** The PPI network. The width of each edge is proportional to the combined score, whereas the color (from light to dark) and size of each node are proportional to interaction strength

Overall, 1216 (4.8%) of the identified 25,298 mRNAs were DEGs, of which 776 were upregulated and 440 were downregulated (Fig. 2a and b, respectively). After removing duplicates, 472 targets of Gly were retrieved from the CTD, STITCH, and GeneCards databases. Venn analysis identified 43 intersection targets (32 upregulated and 11 downregulated; Fig. 2c), which were used to construct a PPI network using the STRING database, which included 43 nodes and 329 edges with an average node degree of 15.3 (Fig. 2d, Additional file 1: Table S1).

### GO and KEGG enrichment analysis

**Fig. 3:**
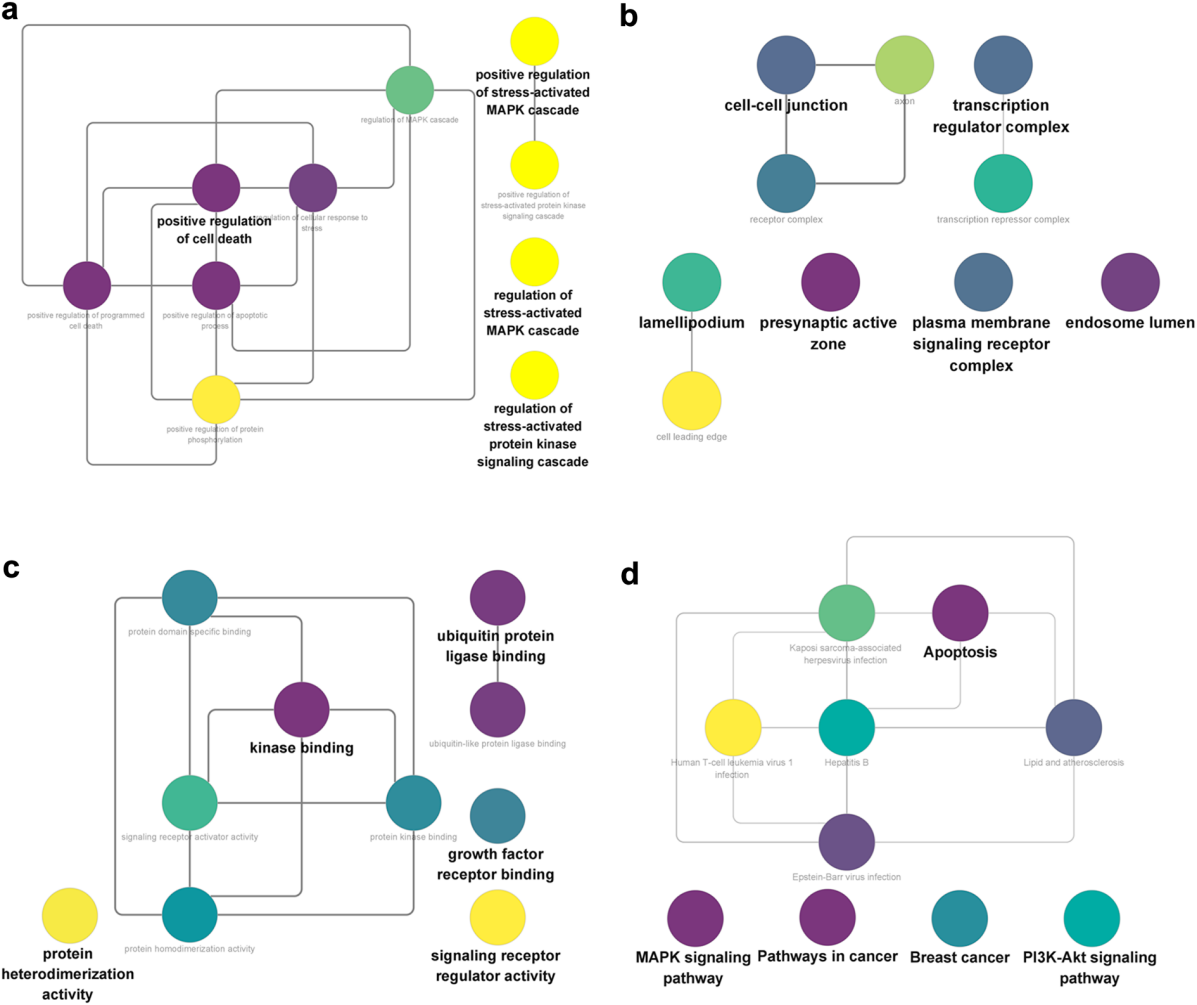
GO and KEGG enrichment analysis. **a** BP enrichment analysis, **b** CC enrichment analysis, **c** MF enrichment analysis, and **d** KEGG pathway enrichment analysis

GO and KEGG pathway enrichment analyses were conducted to investigate the potential mechanisms underlying Gly-induced MDD (Additional file 1: Table S2–3). The overlapping targets were enriched in the BP terms “positive regulation of cell death”, “positive regulation of apoptotic process”, and “regulation of stress-activated MAPK cascade”; the CC terms “presynaptic active zone”, “transcription regulator complex”, and “plasma membrane signaling receptor complex”; and the MF terms “kinase binding”, “growth factor receptor binding”, and “signaling receptor activator activity” (Fig. 3a–c). The KEGG pathway analysis revealed that the common targets were enriched in the terms “MAPK signaling pathway”, “Apoptosis”, and “PI3K-Akt signaling pathway” (Fig. 3d).

### Hub target identification

**Fig. 4:**
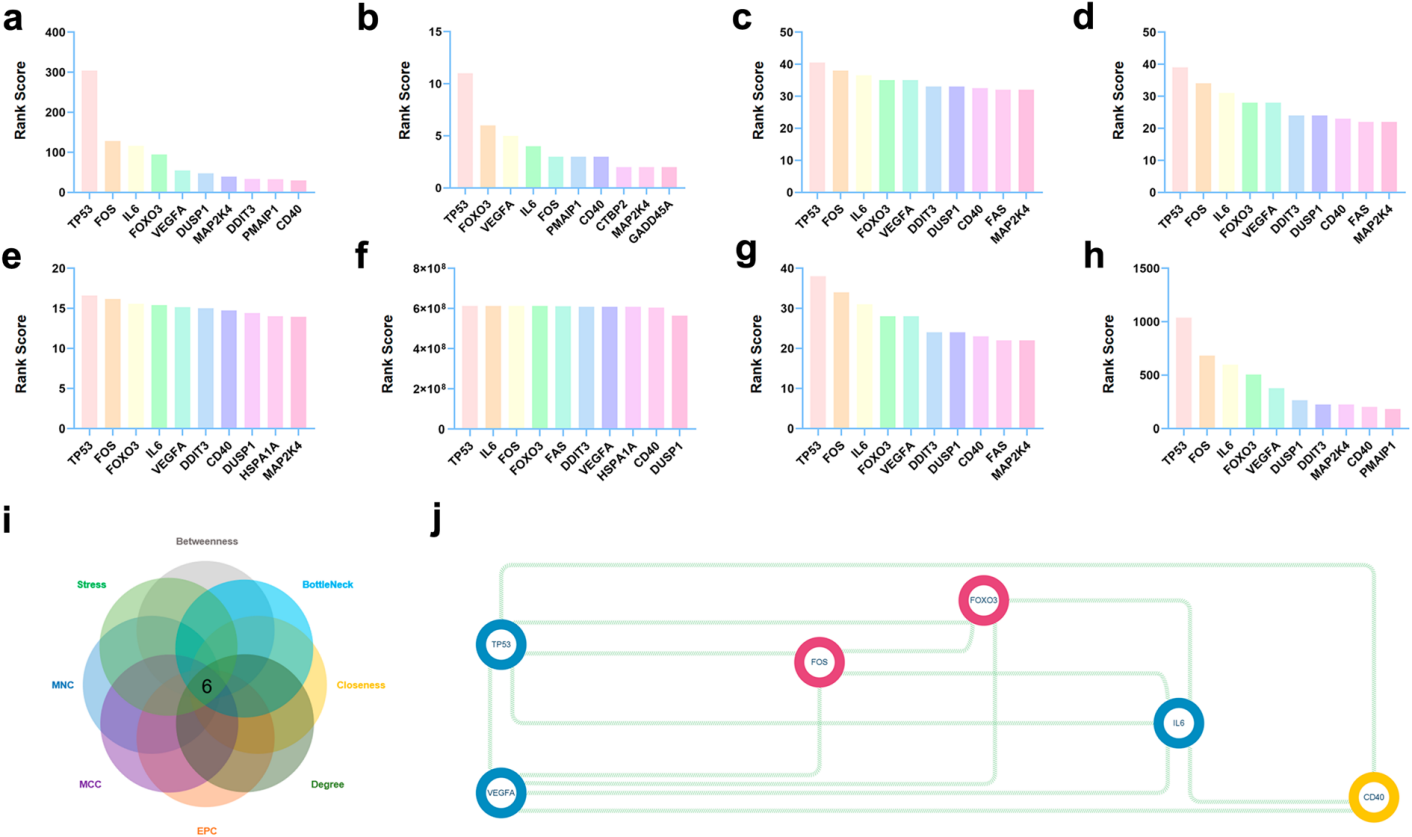
Hub target identification. Top 10 targets calculated through **a** the betweenness method, **b** the bottleneck method, **c** the closeness method, **d** the degree method, **e** the EPC method, and **f** the MCC method, **g** the MNC method, and **h** the stress method. **i** A Venn map of the top 6 core targets. **h** The PPI network of the 6 targets

The top 10 targets were identified using eight topological analysis methods (betweenness, bottleneck, closeness, degree, EPC, MCC, MNC, and stress) (Fig. 4a–h, Additional file 1: Table S4-11). The top six hub targets, as determined by Venn analysis, included cluster of differentiation 40 (CD40), forkhead box O3 (FOXO3), Fos proto-oncogene, AP-1 transcription factor subunit (FOS), interleukin 6 (IL6), tumor protein p53 (TP53), and vascular endothelial growth factor A (VEGFA) (Fig. 4i). PPI networks of the top six hub targets were constructed with the STRING database (Fig. 4j).

### Molecular docking

**Fig. 5:**
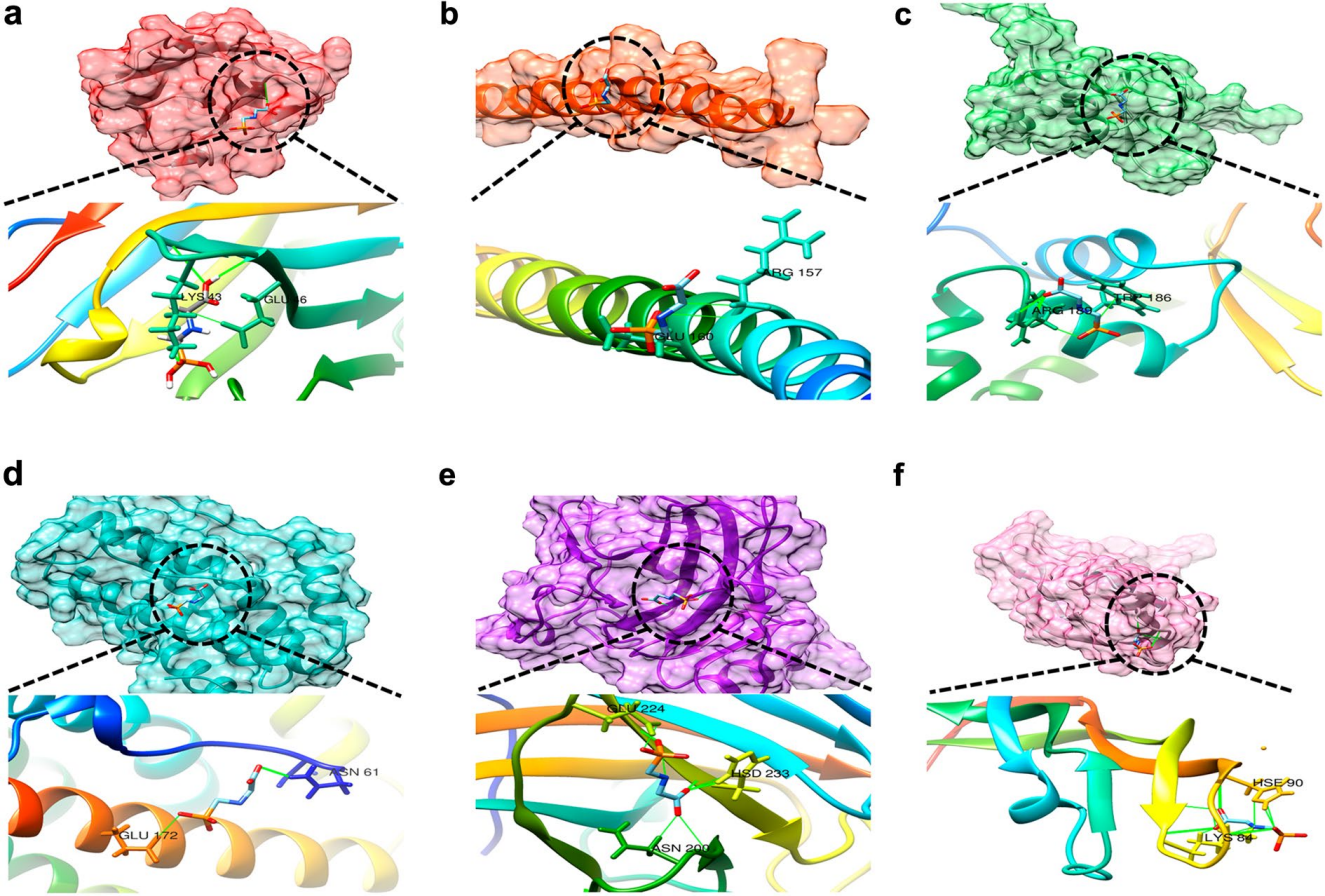
Molecular docking. **a** Gly-CD40, **b** Gly-FOS, **c** Gly-FOXO3, **d** Gly-IL6, **e** Gly-TP53, and **f** Gly-VEGFA

Molecular docking analysis was performed to further investigate the ability of Gly to bind to the top six core targets. The binding energies of Gly with CD40, FOXO3, FOS, IL6, TP53, and VEGFA were − 6.63, − 6.45, − 6.12, − 6.88, − 6.88, and − 6.19 kcal/mol, respectively (Fig. 5a–f).

## Discussion

Environmental factors have been reported to play a significant role in the onset of MDD, as the majority of cases are believed to occur randomly [37]. Epidemiological studies have suggested a possible association between exposure to pesticides and the development of MDD [5]. To better understand the molecular mechanisms underlying Gly-induced MDD, bioinformatics algorithms were used to explore aberrantly expressed mRNAs between MDD patients and controls, which identified 1216 DEGs (776 upregulated and 440 downregulated) from the GSE76826 dataset. Network pharmacology predicted 43 potential targets of Gly associated with the induction of MDD (32 upregulated and 11 downregulated). GO and KEGG analyses were performed to investigate the biological characteristics of these potential targets and six hub targets (CD40, FOXO3, FOS, IL6, TP53, and VEGFA) were identified using molecular docking.

CD40 is a transmembrane protein and a member of the tumor necrosis factor receptor superfamily that plays a crucial role in the initiation and maintenance of inflammatory reactions [16]. CD40-mediated aberrant neuroinflammation can increase permeability of the blood–brain barrier and subsequently damage neurons and glial cells [30]. A previous study suggested that newly diagnosed depression is associated with increased expression of platelet-derived CD40 [29]. In addition, depression-like behavior was associated with abnormal microglial expression of CD40 in a rat model of Alzheimer’s disease [17]. Gly has been demonstrated to induce death of human neuroblastoma SH-SY5Y cells through upregulation of CD40 expression [27]. Therefore, CD40 and related inflammatory signaling pathways are potential targets for the treatment of Gly-induced MDD.

FOXO3 is a crucial transcription factor that regulates various gene networks associated with cellular metabolism that contribute to various physiological and pathological processes [8], and plays a critical role in oxidative stress-induced neuronal death [39]. Recent studies have indicated an association between MDD and selective and persistent death of hippocampal neurons [23]. Although the role of FOXO3 in MDD remains unclear, a prior epidemiological study suggested that genetic variants of FOXO3 may be associated with depressive symptoms in older adults [26]. It has been reported that Gly exposure induced proliferation of breast cancer cells through upregulation of FOXO3 mRNA expression [33]. Collectively, these findings suggest that FOXO3 may play a key role in Gly-induced MDD, although further investigations are needed to clarify the underlying mechanisms.

FOS is a transcription factor that is rapidly and transiently activated [15]. Hence, numerous studies have advocated the use of FOS as an indicator of stimulus-triggered modifications in brain function [15]. Many studies have reported altered expression of FOS in models of depression. Blue light deprivation is reported to produce a depression-like response in Mongolian gerbils accompanied by decreased FOS expression in brain tissues [20]. Another study reported that rice wine can potentially reduce stress-induced depression-like behaviors and FOS expression in rats [28]. In addition, FOS was associated with Gly-induced hematotoxicity via dysregulation of hematopoietic stem cell function [19] and Gly-induced anxiety and depression-like behavior in mice was accompanied by increased FOS expression in brain tissues [1]. However, it remains unclear whether FOS is a key molecule in Gly-induced MDD.

Increased expression of the cytokine IL6, in both peripheral and central systems, is believed to play a vital role in stress reactions and depressive disorders, especially as a comorbidity with physical illnesses [13, 40, 41]. Elevated IL6 activity can potentially induce depression via stimulation of the hypothalamic–pituitary–adrenal axis or modulation of neurotransmitter metabolism [36]. Hence, manipulation of IL-6 activity could benefit individuals with MDD and inflammatory characteristics [36]. Perinatal exposure to Gly significantly increased IL6 expression and caused liver damage in rat offspring [31]. In addition, Gly induced an inflammatory response mediated by elevated IL6 and altered the microbial composition in the rat intestine [34]. Taken together, these findings suggest that IL6 is a potential key molecule in the onset of Gly-induced MDD.

TP53 acts as a transcription factor that regulates the expression of almost 500 target genes that participate in various cellular processes, including cell death, the cell cycle, cell senescence, DNA repair, and metabolism [35]. In addition, TP53 is involved in regulation of the mitogen-activated protein kinase signaling pathway, which plays an important role in the development and progression of MDD [12]. Epidemiological studies have shown that genetic polymorphisms of TP53 are strongly associated with MDD and the minor allele 72 C may be a protective factor against MDD [25]. Gly has been reported to influence TP53 expression in human peripheral blood mononuclear cells by increasing methylation of the TP53 promoter [38]. Recent research of TP53 has mainly focused on tumor formation and development. However, the results of the present study found that TP53 may play a key role in Gly-induced MDD. Therefore, further studies are warranted to investigate the underlying mechanisms.

VEGF is elevated in damaged vessels and neurons, abnormal branching, and chronic inflammatory conditions, such as cardiovascular disease and depression [2, 10]. In a previous study, serum VEGFA mRNA and protein levels were significantly elevated in patients with MDD, suggesting a potentially important role in the pathogenesis of depression [7]. Another study found increased mRNA levels of VEGFA in peripheral blood cells and serum in patients with recurrent depression as compared to healthy controls, and suggested that VEGFA gene polymorphism could be a prognostic factor for the development of recurrent depression [18]. Furthermore, it has been reported that exposure to Gly during pregnancy reduces placental vascular density and cell proliferation via interference with VEGFA expression and subsequent impaired barrier function and nutrient transport in the placenta of newborn piglets [3]. These findings combined with the results of the present study indicate that VEGFA may play an important role in the onset of Gly-induced MDD.

## Conclusion

Network toxicology analyses of the molecular pathways associated with Gly-induced MDD identified CD40, FOXO3, FOS, IL6, TP53, and VEGFA as potential targets. The findings of this preliminary examination of the correlation between exposure to Gly-based herbicides and MDD offer useful references for future research. Nevertheless, further in vivo and in vitro studies are needed to validate these results.

### Supplementary Information

**Additional file 1: Table S1.** Information of PPI network. **Table S2.** GO enrichment analysis. **Table S3.** KEGG enrichment analysis. **Table S4.** Top 10 targets calculated by betweenness method. **Table S5.** Top 10 targets calculated by bottleneck method. **Table S6.** Top 10 targets calculated by closeness method. **Table S7.** Top 10 targets calculated by degree method. **Table S8.** Top 10 targets calculated by EPC method. **Table S9.** Top 10 targets calculated by MCC method. **Table S10.** Top 10 targets calculated by MNC method. **Table S11.** Top 10 targets calculated by stress method

## Data Availability

All data in this study are available from the corresponding authors upon request.
