# Supplementary material for: Clarification of the molecular mechanisms underlying glyphosate-induced major depressive disorder: a network toxicology approach
Source: Ann Gen Psychiatry. 2024 Jan 31;23:8. doi: 10.1186/s12991-024-00491-4 (PMC10829247; doi:10.1186/s12991-024-00491-4)
Supplement: Supplementary file 1 — Additional file 1: Table S1. Information of PPI network. Table S2. GO enrichment analysis. Table S3. KEGG enrichment analysis. Table S4. Top 10 targets calculated by betweenness method. Table S5. Top 10 targets calculated by bottleneck method. Table S6. Top 10 targets calculated by closeness method. Table S7. Top 10 targets calculated by degree method. Table S8. Top 10 targets calculated by EPC method. Table S9. Top 10 targets calculated by MCC method. Table S10. Top 10 targets calculated by MNC method. Table S11. Top 10 targets calculated by stress method [file 12991_2024_491_MOESM1_ESM.docx]

**Supplementary Material**

**Clarification of the Molecular Mechanisms Underlying Glyphosate Induced Major Depressive Disorder: a Network Toxicology Approach**

**Journal name: Behavior Genetics**

**Jianan L^1^ and Haoran Bi^2*^**

1. Department of Occupational and Environmental Health, College of Public Health, Xuzhou Medical University, 209 Tongshan Road, Yun Long District, Xuzhou 221000, China.
2. Department of Biostatistics, College of Public Health, Xuzhou Medical University, 209 Tongshan Road, Yun Long District, Xuzhou 221000, China.

*** Correspondence:** Haoran Bi

**Email：**bihaoran1989@sina.com

**Table S1. Information of PPI network**

| **#node1** | **node2** | **neighborhood_on_chromosome** | **gene_fusion** | **phylogenetic_cooccurrence** | **homology** | **coexpression** | **experimentally_determined_interaction** | **database_annotated** | **automated_textmining** | **combined_score** |
| --- | --- | --- | --- | --- | --- | --- | --- | --- | --- | --- |
| APP | ITGA3 | 0 | 0 | 0 | 0 | 0.102 | 0 | 0 | 0.11 | 0.167 |
| APP | CTSD | 0 | 0 | 0 | 0 | 0.087 | 0.502 | 0 | 0.608 | 0.806 |
| APP | DUSP1 | 0 | 0 | 0 | 0 | 0 | 0 | 0 | 0.165 | 0.165 |
| APP | LMNB1 | 0 | 0 | 0 | 0 | 0.062 | 0 | 0 | 0.167 | 0.185 |
| APP | TP53 | 0 | 0 | 0 | 0 | 0 | 0 | 0 | 0.621 | 0.621 |
| APP | IFNAR1 | 0 | 0 | 0 | 0 | 0 | 0 | 0 | 0.171 | 0.171 |
| APP | WNT7A | 0 | 0 | 0 | 0 | 0.062 | 0 | 0 | 0.143 | 0.162 |
| APP | FAS | 0 | 0 | 0 | 0 | 0 | 0 | 0 | 0.167 | 0.167 |
| APP | GSTM1 | 0 | 0 | 0 | 0 | 0 | 0 | 0 | 0.17 | 0.17 |
| APP | MAP2K4 | 0 | 0 | 0 | 0 | 0 | 0 | 0 | 0.187 | 0.187 |
| APP | DLL1 | 0 | 0 | 0 | 0 | 0 | 0 | 0 | 0.194 | 0.194 |
| APP | CD40 | 0 | 0 | 0 | 0 | 0 | 0 | 0 | 0.243 | 0.242 |
| APP | DDIT3 | 0 | 0 | 0 | 0 | 0 | 0 | 0 | 0.31 | 0.309 |
| APP | CD63 | 0 | 0 | 0 | 0 | 0 | 0 | 0 | 0.323 | 0.323 |
| APP | HSPA1A | 0 | 0 | 0 | 0 | 0 | 0.056 | 0 | 0.336 | 0.347 |
| APP | GAB2 | 0 | 0 | 0 | 0 | 0 | 0 | 0 | 0.411 | 0.411 |
| APP | VEGFA | 0 | 0 | 0 | 0 | 0.067 | 0 | 0 | 0.425 | 0.44 |
| APP | FOS | 0 | 0 | 0 | 0 | 0.062 | 0.063 | 0 | 0.46 | 0.483 |
| APP | IL6 | 0 | 0 | 0 | 0 | 0 | 0 | 0 | 0.619 | 0.619 |
| APP | FOXO3 | 0 | 0 | 0 | 0 | 0.057 | 0 | 0.9 | 0.37 | 0.935 |
| BCL2A1 | DUSP1 | 0 | 0 | 0 | 0 | 0.065 | 0.058 | 0 | 0.34 | 0.367 |
| BCL2A1 | TRAF4 | 0 | 0 | 0 | 0 | 0 | 0 | 0 | 0.262 | 0.262 |
| BCL2A1 | E2F3 | 0 | 0 | 0 | 0 | 0.062 | 0 | 0 | 0.136 | 0.155 |
| BCL2A1 | TNFRSF10C | 0 | 0 | 0 | 0 | 0.087 | 0 | 0 | 0.108 | 0.15 |
| BCL2A1 | DDIT3 | 0 | 0 | 0 | 0 | 0 | 0 | 0 | 0.153 | 0.153 |
| BCL2A1 | HSPA6 | 0 | 0 | 0 | 0 | 0.159 | 0 | 0 | 0 | 0.158 |
| BCL2A1 | HSPA1A | 0 | 0 | 0 | 0 | 0 | 0 | 0 | 0.16 | 0.159 |
| BCL2A1 | FOXO3 | 0 | 0 | 0 | 0 | 0 | 0.057 | 0 | 0.26 | 0.272 |
| BCL2A1 | VEGFA | 0 | 0 | 0 | 0 | 0 | 0 | 0 | 0.324 | 0.323 |
| BCL2A1 | GADD45A | 0 | 0 | 0 | 0 | 0 | 0 | 0 | 0.369 | 0.369 |
| BCL2A1 | FOS | 0 | 0 | 0 | 0 | 0 | 0 | 0 | 0.392 | 0.392 |
| BCL2A1 | FAS | 0 | 0 | 0 | 0 | 0.083 | 0 | 0 | 0.391 | 0.418 |
| BCL2A1 | CD40 | 0 | 0 | 0 | 0 | 0.132 | 0 | 0 | 0.405 | 0.461 |
| BCL2A1 | IL6 | 0 | 0 | 0 | 0 | 0.109 | 0 | 0 | 0.464 | 0.502 |
| BCL2A1 | TP53 | 0 | 0 | 0 | 0 | 0.051 | 0.078 | 0.9 | 0.507 | 0.951 |
| BCL2A1 | PMAIP1 | 0 | 0 | 0 | 0 | 0.062 | 0.904 | 0 | 0.93 | 0.993 |
| CD40 | CTSD | 0 | 0 | 0 | 0 | 0.049 | 0 | 0 | 0.161 | 0.167 |
| CD40 | DUSP1 | 0 | 0 | 0 | 0 | 0.051 | 0 | 0 | 0.28 | 0.287 |
| CD40 | MKNK2 | 0 | 0 | 0 | 0 | 0.065 | 0 | 0 | 0.306 | 0.323 |
| CD40 | TRAF4 | 0 | 0 | 0 | 0 | 0.062 | 0.305 | 0 | 0.617 | 0.729 |
| CD40 | TP53 | 0 | 0 | 0 | 0 | 0.062 | 0 | 0 | 0.464 | 0.475 |
| CD40 | IFNAR1 | 0 | 0 | 0 | 0 | 0 | 0 | 0 | 0.423 | 0.423 |
| CD40 | FOS | 0 | 0 | 0 | 0 | 0 | 0 | 0 | 0.366 | 0.366 |
| CD40 | PMAIP1 | 0 | 0 | 0 | 0 | 0 | 0 | 0 | 0.184 | 0.183 |
| CD40 | FAS | 0 | 0 | 0 | 0 | 0.081 | 0 | 0 | 0.841 | 0.848 |
| CD40 | TNFRSF10C | 0 | 0 | 0 | 0 | 0.062 | 0 | 0 | 0.3 | 0.315 |
| CD40 | MAP3K3 | 0 | 0 | 0 | 0 | 0 | 0.23 | 0 | 0.171 | 0.334 |
| CD40 | DLL1 | 0 | 0 | 0 | 0 | 0 | 0 | 0 | 0.194 | 0.194 |
| CD40 | IL6R | 0 | 0 | 0 | 0 | 0.062 | 0 | 0 | 0.41 | 0.423 |
| CD40 | GADD45A | 0 | 0 | 0 | 0 | 0 | 0 | 0 | 0.184 | 0.183 |
| CD40 | FOXO3 | 0 | 0 | 0 | 0 | 0 | 0 | 0 | 0.21 | 0.21 |
| CD40 | CD63 | 0 | 0 | 0 | 0 | 0 | 0 | 0 | 0.374 | 0.374 |
| CD40 | DDIT3 | 0 | 0 | 0 | 0 | 0 | 0 | 0 | 0.44 | 0.44 |
| CD40 | VEGFA | 0 | 0 | 0 | 0 | 0 | 0 | 0 | 0.56 | 0.56 |
| CD40 | HSPA1A | 0 | 0 | 0 | 0 | 0 | 0.061 | 0 | 0.598 | 0.606 |
| CD40 | IL6 | 0 | 0 | 0 | 0 | 0.11 | 0 | 0 | 0.834 | 0.846 |
| CD40 | MAP2K4 | 0 | 0 | 0 | 0 | 0 | 0.542 | 0.9 | 0.181 | 0.959 |
| CD63 | ITGA3 | 0 | 0 | 0 | 0 | 0 | 0.058 | 0 | 0.369 | 0.38 |
| CD63 | CTSD | 0 | 0 | 0 | 0 | 0.117 | 0 | 0 | 0.539 | 0.576 |
| CD63 | TP53 | 0 | 0 | 0 | 0 | 0 | 0 | 0 | 0.327 | 0.328 |
| CD63 | RALB | 0 | 0 | 0 | 0 | 0.062 | 0 | 0 | 0.137 | 0.156 |
| CD63 | FAS | 0 | 0 | 0 | 0 | 0 | 0 | 0 | 0.184 | 0.183 |
| CD63 | HSPA1A | 0 | 0 | 0 | 0 | 0 | 0 | 0 | 0.387 | 0.387 |
| CD63 | IL6 | 0 | 0 | 0 | 0 | 0 | 0 | 0 | 0.459 | 0.459 |
| CD63 | MAP2K4 | 0 | 0 | 0 | 0 | 0 | 0 | 0 | 0.217 | 0.217 |
| CD63 | VEGFA | 0 | 0 | 0 | 0 | 0 | 0 | 0 | 0.466 | 0.465 |
| CDC25B | DUSP1 | 0 | 0 | 0 | 0 | 0.058 | 0.178 | 0 | 0.338 | 0.443 |
| CDC25B | DLL1 | 0 | 0 | 0 | 0 | 0 | 0.103 | 0 | 0.091 | 0.15 |
| CDC25B | GADD45G | 0 | 0 | 0 | 0 | 0 | 0 | 0 | 0.182 | 0.182 |
| CDC25B | PKN2 | 0 | 0 | 0 | 0 | 0.062 | 0.077 | 0 | 0.138 | 0.188 |
| CDC25B | MAP2K4 | 0 | 0 | 0 | 0 | 0 | 0 | 0 | 0.197 | 0.197 |
| CDC25B | LMNB1 | 0 | 0 | 0 | 0 | 0.101 | 0 | 0 | 0.158 | 0.21 |
| CDC25B | PMAIP1 | 0 | 0 | 0 | 0 | 0 | 0 | 0 | 0.21 | 0.21 |
| CDC25B | PAK1 | 0 | 0 | 0 | 0 | 0.062 | 0.101 | 0 | 0.143 | 0.214 |
| CDC25B | VEGFA | 0 | 0 | 0 | 0 | 0 | 0 | 0 | 0.232 | 0.231 |
| CDC25B | FOS | 0 | 0 | 0 | 0 | 0 | 0.145 | 0 | 0.182 | 0.271 |
| CDC25B | FOXO3 | 0 | 0 | 0 | 0 | 0.064 | 0.105 | 0 | 0.261 | 0.327 |
| CDC25B | GADD45A | 0 | 0 | 0 | 0 | 0 | 0 | 0 | 0.378 | 0.378 |
| CDC25B | E2F3 | 0 | 0 | 0 | 0 | 0.063 | 0 | 0 | 0.406 | 0.42 |
| CDC25B | TP53 | 0 | 0 | 0 | 0 | 0.063 | 0 | 0 | 0.538 | 0.548 |
| CREB5 | DUSP1 | 0 | 0 | 0 | 0 | 0.063 | 0.139 | 0 | 0.062 | 0.177 |
| CREB5 | TRAF4 | 0 | 0 | 0 | 0 | 0 | 0.244 | 0 | 0.101 | 0.291 |
| CREB5 | E2F3 | 0 | 0 | 0 | 0 | 0 | 0 | 0 | 0.185 | 0.185 |
| CREB5 | TP53 | 0 | 0 | 0 | 0 | 0 | 0 | 0 | 0.18 | 0.179 |
| CREB5 | FOS | 0 | 0 | 0 | 0 | 0 | 0.876 | 0 | 0.235 | 0.901 |
| CREB5 | IL6R | 0 | 0 | 0 | 0 | 0 | 0 | 0 | 0.165 | 0.165 |
| CREB5 | FZD3 | 0 | 0 | 0 | 0 | 0 | 0.062 | 0 | 0.178 | 0.196 |
| CREB5 | DDIT3 | 0 | 0 | 0 | 0 | 0 | 0.078 | 0 | 0.183 | 0.214 |
| CREB5 | GADD45A | 0 | 0 | 0 | 0 | 0.049 | 0 | 0 | 0.216 | 0.222 |
| CTBP2 | LPO | 0 | 0 | 0 | 0 | 0 | 0.175 | 0 | 0 | 0.175 |
| CTBP2 | E2F3 | 0 | 0 | 0 | 0 | 0.062 | 0 | 0 | 0.131 | 0.15 |
| CTBP2 | TP53 | 0 | 0 | 0 | 0 | 0 | 0 | 0 | 0.26 | 0.26 |
| CTBP2 | PAK1 | 0 | 0 | 0 | 0 | 0.064 | 0 | 0 | 0.234 | 0.253 |
| CTBP2 | WNT7A | 0 | 0 | 0 | 0 | 0 | 0.077 | 0 | 0.169 | 0.2 |
| CTBP2 | FOS | 0 | 0 | 0 | 0 | 0 | 0 | 0 | 0.166 | 0.165 |
| CTBP2 | GSTM1 | 0 | 0 | 0 | 0 | 0 | 0.152 | 0 | 0.083 | 0.189 |
| CTBP2 | PMAIP1 | 0 | 0 | 0 | 0 | 0 | 0 | 0 | 0.17 | 0.169 |
| CTBP2 | FOXO3 | 0 | 0 | 0 | 0 | 0 | 0.149 | 0 | 0.088 | 0.191 |
| CTBP2 | DLL1 | 0 | 0 | 0 | 0 | 0 | 0.065 | 0 | 0.204 | 0.224 |
| CTSD | MAP2K4 | 0 | 0 | 0 | 0 | 0 | 0 | 0 | 0.151 | 0.151 |
| CTSD | LMNB1 | 0 | 0 | 0 | 0 | 0.062 | 0 | 0 | 0.159 | 0.178 |
| CTSD | HSPA6 | 0 | 0 | 0 | 0 | 0.086 | 0.078 | 0 | 0.118 | 0.192 |
| CTSD | DDIT3 | 0 | 0 | 0 | 0 | 0 | 0 | 0 | 0.194 | 0.194 |
| CTSD | LPO | 0 | 0 | 0 | 0 | 0.089 | 0 | 0 | 0.159 | 0.201 |
| CTSD | FOXO3 | 0 | 0 | 0 | 0 | 0 | 0 | 0 | 0.212 | 0.212 |
| CTSD | GSTM1 | 0 | 0 | 0 | 0 | 0.062 | 0.132 | 0 | 0.149 | 0.247 |
| CTSD | FAS | 0 | 0 | 0 | 0 | 0 | 0 | 0 | 0.279 | 0.279 |
| CTSD | FOS | 0 | 0 | 0 | 0 | 0 | 0.056 | 0 | 0.284 | 0.295 |
| CTSD | GAB2 | 0 | 0 | 0 | 0 | 0 | 0 | 0 | 0.304 | 0.304 |
| CTSD | HSPA1A | 0 | 0 | 0 | 0 | 0.097 | 0.078 | 0 | 0.238 | 0.31 |
| CTSD | IL6 | 0 | 0 | 0 | 0 | 0 | 0 | 0 | 0.391 | 0.39 |
| CTSD | VEGFA | 0 | 0 | 0 | 0 | 0 | 0 | 0 | 0.405 | 0.405 |
| CTSD | TP53 | 0 | 0 | 0 | 0 | 0 | 0 | 0 | 0.518 | 0.518 |
| DDIT3 | DUSP1 | 0 | 0 | 0 | 0 | 0.062 | 0 | 0 | 0.325 | 0.339 |
| DDIT3 | MKNK2 | 0 | 0 | 0 | 0 | 0.062 | 0 | 0 | 0.138 | 0.156 |
| DDIT3 | GADD45G | 0 | 0 | 0 | 0 | 0 | 0 | 0 | 0.502 | 0.502 |
| DDIT3 | LMNB1 | 0 | 0 | 0 | 0 | 0 | 0 | 0 | 0.176 | 0.176 |
| DDIT3 | E2F3 | 0 | 0 | 0 | 0 | 0 | 0.11 | 0 | 0.107 | 0.171 |
| DDIT3 | TP53 | 0 | 0 | 0 | 0 | 0 | 0 | 0 | 0.603 | 0.603 |
| DDIT3 | IFNAR1 | 0 | 0 | 0 | 0 | 0 | 0 | 0 | 0.153 | 0.153 |
| DDIT3 | FOS | 0 | 0 | 0 | 0 | 0 | 0.875 | 0 | 0.472 | 0.931 |
| DDIT3 | HSPA6 | 0 | 0 | 0 | 0 | 0 | 0 | 0 | 0.391 | 0.39 |
| DDIT3 | PMAIP1 | 0 | 0 | 0 | 0 | 0 | 0 | 0 | 0.505 | 0.505 |
| DDIT3 | FAS | 0 | 0 | 0 | 0 | 0 | 0 | 0 | 0.396 | 0.396 |
| DDIT3 | TNFRSF10C | 0 | 0 | 0 | 0 | 0 | 0 | 0 | 0.173 | 0.173 |
| DDIT3 | IL6R | 0 | 0 | 0 | 0 | 0 | 0 | 0 | 0.163 | 0.163 |
| DDIT3 | GADD45A | 0 | 0 | 0 | 0 | 0.062 | 0 | 0 | 0.661 | 0.668 |
| DDIT3 | HSPA1A | 0 | 0 | 0 | 0 | 0 | 0 | 0 | 0.474 | 0.474 |
| DDIT3 | IL6 | 0 | 0 | 0 | 0 | 0 | 0 | 0 | 0.535 | 0.535 |
| DDIT3 | FOXO3 | 0 | 0 | 0 | 0 | 0 | 0.078 | 0 | 0.327 | 0.353 |
| DDIT3 | MAP2K4 | 0 | 0 | 0 | 0 | 0 | 0 | 0 | 0.231 | 0.231 |
| DDIT3 | VEGFA | 0 | 0 | 0 | 0 | 0 | 0 | 0 | 0.392 | 0.392 |
| DLL1 | GADD45G | 0 | 0 | 0 | 0 | 0.091 | 0 | 0 | 0.119 | 0.164 |
| DLL1 | E2F3 | 0 | 0 | 0 | 0 | 0.064 | 0 | 0 | 0.236 | 0.255 |
| DLL1 | TP53 | 0 | 0 | 0 | 0 | 0 | 0 | 0 | 0.392 | 0.392 |
| DLL1 | RALB | 0 | 0 | 0 | 0 | 0 | 0.139 | 0 | 0.078 | 0.172 |
| DLL1 | WNT7A | 0 | 0 | 0 | 0 | 0 | 0.108 | 0 | 0.313 | 0.361 |
| DLL1 | FOS | 0 | 0 | 0 | 0 | 0 | 0 | 0 | 0.171 | 0.171 |
| DLL1 | IL6R | 0 | 0 | 0 | 0 | 0 | 0 | 0 | 0.168 | 0.168 |
| DLL1 | FOXO3 | 0 | 0 | 0 | 0 | 0 | 0 | 0 | 0.18 | 0.179 |
| DLL1 | FZD3 | 0 | 0 | 0 | 0 | 0 | 0.074 | 0 | 0.205 | 0.232 |
| DLL1 | IL6 | 0 | 0 | 0 | 0 | 0 | 0 | 0 | 0.37 | 0.37 |
| DLL1 | VEGFA | 0 | 0 | 0 | 0 | 0.062 | 0 | 0 | 0.477 | 0.489 |
| DUSP1 | IL6R | 0 | 0 | 0 | 0 | 0 | 0 | 0 | 0.156 | 0.155 |
| DUSP1 | FAS | 0 | 0 | 0 | 0 | 0 | 0 | 0 | 0.17 | 0.169 |
| DUSP1 | PKN2 | 0 | 0 | 0 | 0 | 0.052 | 0.151 | 0 | 0.058 | 0.175 |
| DUSP1 | TRAF4 | 0 | 0 | 0 | 0 | 0 | 0.061 | 0 | 0.161 | 0.178 |
| DUSP1 | PMAIP1 | 0 | 0 | 0 | 0 | 0 | 0 | 0 | 0.185 | 0.185 |
| DUSP1 | PAK1 | 0 | 0 | 0 | 0 | 0 | 0.15 | 0 | 0.101 | 0.203 |
| DUSP1 | FOXO3 | 0 | 0 | 0 | 0 | 0 | 0.09 | 0 | 0.261 | 0.299 |
| DUSP1 | HSPA6 | 0 | 0 | 0 | 0 | 0.076 | 0.058 | 0 | 0.266 | 0.306 |
| DUSP1 | GADD45G | 0 | 0 | 0 | 0 | 0.08 | 0 | 0 | 0.307 | 0.335 |
| DUSP1 | MAP3K3 | 0 | 0 | 0 | 0 | 0 | 0.157 | 0 | 0.29 | 0.376 |
| DUSP1 | MAP2K4 | 0 | 0 | 0 | 0 | 0 | 0 | 0 | 0.39 | 0.39 |
| DUSP1 | VEGFA | 0 | 0 | 0 | 0 | 0.062 | 0 | 0 | 0.393 | 0.406 |
| DUSP1 | GADD45A | 0 | 0 | 0 | 0 | 0.062 | 0 | 0 | 0.394 | 0.407 |
| DUSP1 | MKNK2 | 0 | 0 | 0 | 0 | 0 | 0 | 0 | 0.443 | 0.443 |
| DUSP1 | TP53 | 0 | 0 | 0 | 0 | 0 | 0.059 | 0 | 0.5 | 0.509 |
| DUSP1 | IL6 | 0 | 0 | 0 | 0 | 0.108 | 0 | 0 | 0.567 | 0.597 |
| DUSP1 | HSPA1A | 0 | 0 | 0 | 0 | 0.074 | 0.058 | 0.9 | 0.347 | 0.935 |
| DUSP1 | FOS | 0 | 0 | 0 | 0 | 0.872 | 0.139 | 0 | 0.686 | 0.962 |
| E2F3 | GADD45G | 0 | 0 | 0 | 0 | 0 | 0 | 0 | 0.227 | 0.227 |
| E2F3 | LMNB1 | 0 | 0 | 0 | 0 | 0.109 | 0 | 0 | 0.15 | 0.21 |
| E2F3 | MAP2K4 | 0 | 0 | 0 | 0 | 0 | 0 | 0 | 0.177 | 0.177 |
| E2F3 | IL6 | 0 | 0 | 0 | 0 | 0 | 0 | 0 | 0.212 | 0.212 |
| E2F3 | PMAIP1 | 0 | 0 | 0 | 0 | 0.076 | 0 | 0 | 0.207 | 0.235 |
| E2F3 | VEGFA | 0 | 0 | 0 | 0 | 0 | 0 | 0 | 0.327 | 0.327 |
| E2F3 | GADD45A | 0 | 0 | 0 | 0 | 0 | 0 | 0 | 0.341 | 0.341 |
| E2F3 | FOS | 0 | 0 | 0 | 0 | 0 | 0 | 0 | 0.37 | 0.37 |
| E2F3 | FOXO3 | 0 | 0 | 0 | 0 | 0 | 0.131 | 0 | 0.306 | 0.371 |
| E2F3 | TP53 | 0 | 0 | 0 | 0 | 0.062 | 0 | 0 | 0.681 | 0.688 |
| FAS | ITGA3 | 0 | 0 | 0 | 0 | 0 | 0 | 0 | 0.152 | 0.152 |
| FAS | LMNB1 | 0 | 0 | 0 | 0 | 0 | 0 | 0 | 0.203 | 0.203 |
| FAS | TRAF4 | 0 | 0 | 0 | 0 | 0 | 0 | 0 | 0.184 | 0.183 |
| FAS | TP53 | 0 | 0 | 0 | 0 | 0.063 | 0 | 0.9 | 0.682 | 0.967 |
| FAS | IFNAR1 | 0 | 0 | 0 | 0 | 0 | 0 | 0 | 0.251 | 0.251 |
| FAS | FOS | 0 | 0 | 0 | 0 | 0 | 0 | 0 | 0.273 | 0.273 |
| FAS | PMAIP1 | 0 | 0 | 0 | 0 | 0 | 0 | 0 | 0.56 | 0.56 |
| FAS | HSPA1A | 0 | 0 | 0 | 0 | 0 | 0 | 0 | 0.208 | 0.208 |
| FAS | FOXO3 | 0 | 0 | 0 | 0 | 0 | 0 | 0 | 0.282 | 0.282 |
| FAS | IL6R | 0 | 0 | 0 | 0 | 0.062 | 0 | 0 | 0.297 | 0.312 |
| FAS | GADD45A | 0 | 0 | 0 | 0 | 0 | 0.111 | 0 | 0.322 | 0.371 |
| FAS | VEGFA | 0 | 0 | 0 | 0 | 0 | 0 | 0 | 0.421 | 0.421 |
| FAS | TNFRSF10C | 0 | 0 | 0 | 0 | 0.069 | 0 | 0 | 0.54 | 0.554 |
| FAS | IL6 | 0 | 0 | 0 | 0 | 0.083 | 0 | 0 | 0.602 | 0.619 |
| FAS | MAP2K4 | 0 | 0 | 0 | 0 | 0 | 0 | 0.9 | 0.194 | 0.915 |
| FOS | MKNK2 | 0 | 0 | 0 | 0 | 0.062 | 0 | 0 | 0.226 | 0.243 |
| FOS | GADD45G | 0 | 0 | 0 | 0 | 0.062 | 0 | 0 | 0.323 | 0.337 |
| FOS | LMNB1 | 0 | 0 | 0 | 0 | 0 | 0 | 0 | 0.487 | 0.487 |
| FOS | TRAF4 | 0 | 0 | 0 | 0 | 0 | 0.244 | 0 | 0.287 | 0.438 |
| FOS | TP53 | 0 | 0 | 0 | 0 | 0 | 0 | 0.6 | 0.717 | 0.882 |
| FOS | IFNAR1 | 0 | 0 | 0 | 0 | 0 | 0 | 0 | 0.195 | 0.195 |
| FOS | PAK1 | 0 | 0 | 0 | 0 | 0 | 0.06 | 0 | 0.162 | 0.179 |
| FOS | WNT7A | 0 | 0 | 0 | 0 | 0 | 0 | 0 | 0.246 | 0.245 |
| FOS | INHBB | 0 | 0 | 0 | 0 | 0 | 0 | 0 | 0.161 | 0.161 |
| FOS | FZD3 | 0 | 0 | 0 | 0 | 0 | 0.062 | 0 | 0.136 | 0.155 |
| FOS | GSTM1 | 0 | 0 | 0 | 0 | 0 | 0 | 0 | 0.174 | 0.175 |
| FOS | MAP3K3 | 0 | 0 | 0 | 0 | 0 | 0.061 | 0 | 0.177 | 0.194 |
| FOS | IL6R | 0 | 0 | 0 | 0 | 0 | 0 | 0 | 0.247 | 0.246 |
| FOS | PMAIP1 | 0 | 0 | 0 | 0 | 0 | 0 | 0 | 0.276 | 0.276 |
| FOS | GAB2 | 0 | 0 | 0 | 0 | 0 | 0 | 0 | 0.28 | 0.28 |
| FOS | HSPA6 | 0 | 0 | 0 | 0 | 0.052 | 0.058 | 0 | 0.271 | 0.292 |
| FOS | MAP2K4 | 0 | 0 | 0 | 0 | 0 | 0 | 0 | 0.477 | 0.477 |
| FOS | GADD45A | 0 | 0 | 0 | 0 | 0.062 | 0 | 0 | 0.47 | 0.481 |
| FOS | HSPA1A | 0 | 0 | 0 | 0 | 0.09 | 0.079 | 0 | 0.466 | 0.514 |
| FOS | VEGFA | 0 | 0 | 0 | 0 | 0 | 0.273 | 0 | 0.602 | 0.698 |
| FOS | FOXO3 | 0 | 0 | 0 | 0 | 0 | 0.182 | 0.9 | 0.414 | 0.947 |
| FOS | IL6 | 0 | 0 | 0 | 0 | 0.088 | 0 | 0.9 | 0.707 | 0.97 |
| FOXO3 | GADD45G | 0 | 0 | 0 | 0 | 0 | 0 | 0 | 0.203 | 0.203 |
| FOXO3 | LMNB1 | 0 | 0 | 0 | 0 | 0 | 0.132 | 0 | 0.374 | 0.433 |
| FOXO3 | TRAF4 | 0 | 0 | 0 | 0 | 0 | 0 | 0 | 0.208 | 0.208 |
| FOXO3 | TP53 | 0 | 0 | 0 | 0 | 0 | 0.808 | 0 | 0.988 | 0.997 |
| FOXO3 | PAK1 | 0 | 0 | 0 | 0 | 0 | 0.069 | 0 | 0.164 | 0.188 |
| FOXO3 | WNT7A | 0 | 0 | 0 | 0 | 0 | 0.065 | 0 | 0.154 | 0.176 |
| FOXO3 | PMAIP1 | 0 | 0 | 0 | 0 | 0 | 0 | 0 | 0.519 | 0.519 |
| FOXO3 | MAP3K3 | 0 | 0 | 0 | 0 | 0 | 0.071 | 0 | 0.123 | 0.15 |
| FOXO3 | GAB2 | 0 | 0 | 0 | 0 | 0 | 0 | 0 | 0.16 | 0.159 |
| FOXO3 | IL6R | 0 | 0 | 0 | 0 | 0 | 0 | 0 | 0.183 | 0.183 |
| FOXO3 | THEM4 | 0 | 0 | 0 | 0 | 0 | 0 | 0 | 0.171 | 0.171 |
| FOXO3 | GADD45A | 0 | 0 | 0 | 0 | 0.062 | 0 | 0.9 | 0.636 | 0.962 |
| FOXO3 | HSPA1A | 0 | 0 | 0 | 0 | 0 | 0.056 | 0 | 0.337 | 0.348 |
| FOXO3 | IL6 | 0 | 0 | 0 | 0 | 0 | 0.07 | 0 | 0.548 | 0.562 |
| FOXO3 | MAP2K4 | 0 | 0 | 0 | 0 | 0 | 0 | 0 | 0.308 | 0.308 |
| FOXO3 | VEGFA | 0 | 0 | 0 | 0 | 0 | 0 | 0 | 0.504 | 0.504 |
| FZD3 | TP53 | 0 | 0 | 0 | 0 | 0 | 0 | 0 | 0.18 | 0.179 |
| FZD3 | WNT7A | 0 | 0 | 0 | 0 | 0.062 | 0.166 | 0.6 | 0.63 | 0.869 |
| FZD3 | MAP2K4 | 0 | 0 | 0 | 0 | 0 | 0 | 0 | 0.177 | 0.177 |
| GAB2 | TP53 | 0 | 0 | 0 | 0 | 0 | 0 | 0 | 0.241 | 0.24 |
| GAB2 | IFNAR1 | 0 | 0 | 0 | 0 | 0 | 0 | 0 | 0.462 | 0.462 |
| GAB2 | VEGFA | 0 | 0 | 0 | 0 | 0 | 0 | 0 | 0.193 | 0.193 |
| GAB2 | THEM4 | 0 | 0 | 0 | 0 | 0 | 0 | 0 | 0.414 | 0.414 |
| GAB2 | IL6R | 0 | 0 | 0 | 0 | 0 | 0 | 0.9 | 0.061 | 0.902 |
| GAB2 | IL6 | 0 | 0 | 0 | 0 | 0 | 0 | 0.9 | 0.171 | 0.913 |
| GADD45A | GADD45G | 0 | 0 | 0 | 0.948 | 0.063 | 0.27 | 0.9 | 0.808 | 0.928 |
| GADD45A | LMNB1 | 0 | 0 | 0 | 0 | 0 | 0 | 0 | 0.212 | 0.212 |
| GADD45A | TRAF4 | 0 | 0 | 0 | 0 | 0 | 0 | 0 | 0.238 | 0.238 |
| GADD45A | TP53 | 0 | 0 | 0 | 0 | 0.063 | 0.046 | 0.9 | 0.925 | 0.992 |
| GADD45A | HSPA6 | 0 | 0 | 0 | 0 | 0 | 0 | 0 | 0.296 | 0.296 |
| GADD45A | PMAIP1 | 0 | 0 | 0 | 0 | 0.062 | 0 | 0 | 0.633 | 0.641 |
| GADD45A | MAP2K4 | 0 | 0 | 0 | 0 | 0 | 0 | 0 | 0.257 | 0.256 |
| GADD45A | VEGFA | 0 | 0 | 0 | 0 | 0.065 | 0 | 0 | 0.314 | 0.331 |
| GADD45A | HSPA1A | 0 | 0 | 0 | 0 | 0 | 0 | 0 | 0.338 | 0.338 |
| GADD45A | IL6 | 0 | 0 | 0 | 0 | 0.058 | 0 | 0 | 0.37 | 0.381 |
| GADD45G | INHBB | 0 | 0 | 0 | 0 | 0 | 0 | 0 | 0.163 | 0.163 |
| GADD45G | IFNAR1 | 0 | 0 | 0 | 0 | 0 | 0 | 0 | 0.202 | 0.202 |
| GADD45G | MAP2K4 | 0 | 0 | 0 | 0 | 0 | 0 | 0 | 0.216 | 0.216 |
| GADD45G | HSPA1A | 0 | 0 | 0 | 0 | 0.06 | 0 | 0 | 0.203 | 0.218 |
| GADD45G | IL6 | 0 | 0 | 0 | 0 | 0 | 0 | 0 | 0.218 | 0.218 |
| GADD45G | HSPA6 | 0 | 0 | 0 | 0 | 0 | 0 | 0 | 0.236 | 0.236 |
| GADD45G | PMAIP1 | 0 | 0 | 0 | 0 | 0.062 | 0 | 0 | 0.224 | 0.24 |
| GADD45G | TP53 | 0 | 0 | 0 | 0 | 0.052 | 0 | 0.8 | 0.575 | 0.912 |
| GSTM1 | TP53 | 0 | 0 | 0 | 0 | 0 | 0 | 0 | 0.556 | 0.556 |
| GSTM1 | PAK1 | 0 | 0 | 0 | 0 | 0 | 0 | 0 | 0.183 | 0.183 |
| GSTM1 | HSPA6 | 0 | 0 | 0 | 0 | 0.062 | 0.158 | 0 | 0.067 | 0.198 |
| GSTM1 | VEGFA | 0 | 0 | 0 | 0 | 0 | 0 | 0 | 0.244 | 0.243 |
| GSTM1 | HSPA1A | 0 | 0 | 0 | 0 | 0.062 | 0.158 | 0 | 0.2 | 0.312 |
| GSTM1 | IL6 | 0 | 0 | 0 | 0 | 0 | 0 | 0 | 0.359 | 0.359 |
| HSPA1A | LMNB1 | 0 | 0 | 0 | 0 | 0 | 0 | 0 | 0.154 | 0.154 |
| HSPA1A | TP53 | 0 | 0 | 0 | 0 | 0 | 0.752 | 0.9 | 0.955 | 0.998 |
| HSPA1A | HSPA6 | 0 | 0 | 0.449 | 0.982 | 0.117 | 0.675 | 0.6 | 0.742 | 0.877 |
| HSPA1A | PMAIP1 | 0 | 0 | 0 | 0 | 0 | 0 | 0 | 0.263 | 0.263 |
| HSPA1A | MAP3K3 | 0 | 0 | 0 | 0 | 0 | 0.14 | 0 | 0.063 | 0.161 |
| HSPA1A | MAP2K4 | 0 | 0 | 0 | 0 | 0 | 0 | 0 | 0.181 | 0.181 |
| HSPA1A | VEGFA | 0 | 0 | 0 | 0 | 0 | 0 | 0 | 0.324 | 0.324 |
| HSPA1A | IL6 | 0 | 0 | 0 | 0 | 0 | 0 | 0 | 0.532 | 0.532 |
| HSPA6 | TP53 | 0 | 0 | 0 | 0 | 0 | 0.145 | 0 | 0.27 | 0.349 |
| HSPA6 | IL6 | 0 | 0 | 0 | 0 | 0.069 | 0 | 0 | 0.163 | 0.187 |
| IFNAR1 | TP53 | 0 | 0 | 0 | 0 | 0 | 0 | 0 | 0.261 | 0.261 |
| IFNAR1 | PMAIP1 | 0 | 0 | 0 | 0 | 0 | 0 | 0 | 0.163 | 0.163 |
| IFNAR1 | VEGFA | 0 | 0 | 0 | 0 | 0 | 0 | 0 | 0.212 | 0.212 |
| IFNAR1 | IL6R | 0 | 0 | 0 | 0 | 0.062 | 0 | 0 | 0.341 | 0.355 |
| IFNAR1 | IL6 | 0 | 0 | 0 | 0 | 0 | 0 | 0.6 | 0.611 | 0.838 |
| IL6 | ITGA3 | 0 | 0 | 0 | 0 | 0 | 0 | 0 | 0.164 | 0.163 |
| IL6 | LMNB1 | 0 | 0 | 0 | 0 | 0 | 0 | 0 | 0.353 | 0.353 |
| IL6 | LPO | 0 | 0 | 0 | 0 | 0.062 | 0 | 0 | 0.169 | 0.188 |
| IL6 | TRAF4 | 0 | 0 | 0 | 0 | 0 | 0 | 0 | 0.298 | 0.298 |
| IL6 | TP53 | 0 | 0 | 0 | 0 | 0.062 | 0 | 0 | 0.738 | 0.744 |
| IL6 | WNT7A | 0 | 0 | 0 | 0 | 0 | 0 | 0 | 0.292 | 0.292 |
| IL6 | PMAIP1 | 0 | 0 | 0 | 0 | 0 | 0 | 0 | 0.325 | 0.325 |
| IL6 | TNFRSF10C | 0 | 0 | 0 | 0 | 0.056 | 0 | 0 | 0.198 | 0.211 |
| IL6 | MAP3K3 | 0 | 0 | 0 | 0 | 0 | 0 | 0 | 0.242 | 0.241 |
| IL6 | IL6R | 0 | 0 | 0 | 0 | 0 | 0.978 | 0.9 | 0.989 | 0.999 |
| IL6 | MAP2K4 | 0 | 0 | 0 | 0 | 0 | 0 | 0 | 0.338 | 0.338 |
| IL6 | VEGFA | 0 | 0 | 0 | 0 | 0.063 | 0 | 0.9 | 0.879 | 0.987 |
| IL6R | LMNB1 | 0 | 0 | 0 | 0 | 0 | 0 | 0 | 0.171 | 0.171 |
| IL6R | TP53 | 0 | 0 | 0 | 0 | 0 | 0 | 0 | 0.329 | 0.329 |
| IL6R | VEGFA | 0 | 0 | 0 | 0 | 0 | 0 | 0 | 0.47 | 0.47 |
| INHBB | LMNB1 | 0 | 0 | 0 | 0 | 0 | 0.213 | 0 | 0 | 0.213 |
| INHBB | TP53 | 0 | 0 | 0 | 0 | 0 | 0 | 0 | 0.173 | 0.173 |
| INHBB | RALB | 0 | 0 | 0 | 0 | 0 | 0.057 | 0 | 0.281 | 0.292 |
| INHBB | WNT7A | 0 | 0 | 0 | 0 | 0.049 | 0.145 | 0 | 0.084 | 0.19 |
| ITGA3 | MAP3K3 | 0 | 0 | 0 | 0 | 0 | 0 | 0 | 0.177 | 0.177 |
| ITGA3 | TP53 | 0 | 0 | 0 | 0 | 0 | 0 | 0 | 0.308 | 0.307 |
| ITGA3 | VEGFA | 0 | 0 | 0 | 0 | 0.068 | 0 | 0.9 | 0.276 | 0.926 |
| LMNB1 | TRAF4 | 0 | 0 | 0 | 0 | 0.109 | 0 | 0 | 0.094 | 0.159 |
| LMNB1 | PMAIP1 | 0 | 0 | 0 | 0 | 0.088 | 0 | 0 | 0.15 | 0.191 |
| LMNB1 | VEGFA | 0 | 0 | 0 | 0 | 0 | 0 | 0 | 0.214 | 0.214 |
| LMNB1 | TP53 | 0 | 0 | 0 | 0 | 0.086 | 0 | 0 | 0.518 | 0.54 |
| MAP2K4 | TRAF4 | 0 | 0 | 0 | 0 | 0 | 0 | 0 | 0.307 | 0.307 |
| MAP2K4 | TP53 | 0 | 0 | 0 | 0 | 0 | 0 | 0 | 0.561 | 0.561 |
| MAP2K4 | PAK1 | 0 | 0 | 0.306 | 0.67 | 0.062 | 0.25 | 0.9 | 0.181 | 0.933 |
| MAP2K4 | MAP3K3 | 0 | 0 | 0 | 0.627 | 0 | 0.523 | 0.9 | 0.484 | 0.958 |
| MAP2K4 | PKN2 | 0 | 0 | 0 | 0.587 | 0 | 0.118 | 0 | 0.181 | 0.171 |
| MAP2K4 | VEGFA | 0 | 0 | 0 | 0 | 0 | 0 | 0 | 0.261 | 0.261 |
| MAP3K3 | TRAF4 | 0 | 0 | 0 | 0 | 0 | 0.057 | 0 | 0.808 | 0.812 |
| MAP3K3 | TP53 | 0 | 0 | 0 | 0 | 0 | 0 | 0 | 0.242 | 0.241 |
| MAP3K3 | RALB | 0 | 0 | 0 | 0 | 0.054 | 0.213 | 0 | 0.089 | 0.262 |
| MAP3K3 | PAK1 | 0 | 0 | 0 | 0.662 | 0.052 | 0.343 | 0.8 | 0.317 | 0.877 |
| MAP3K3 | VEGFA | 0 | 0 | 0 | 0 | 0.053 | 0 | 0 | 0.257 | 0.266 |
| MAP3K3 | PKN2 | 0 | 0 | 0 | 0.578 | 0.065 | 0.252 | 0 | 0.198 | 0.321 |
| MKNK2 | TP53 | 0 | 0 | 0 | 0 | 0 | 0 | 0 | 0.181 | 0.181 |
| PAK1 | TP53 | 0 | 0 | 0 | 0 | 0 | 0 | 0 | 0.26 | 0.26 |
| PAK1 | RALB | 0 | 0 | 0 | 0 | 0.062 | 0.18 | 0 | 0.099 | 0.246 |
| PAK1 | PKN2 | 0 | 0 | 0 | 0.61 | 0.063 | 0.115 | 0 | 0.234 | 0.203 |
| PAK1 | VEGFA | 0 | 0 | 0 | 0 | 0 | 0 | 0 | 0.246 | 0.245 |
| PALLD | TP53 | 0 | 0 | 0 | 0 | 0 | 0 | 0 | 0.194 | 0.194 |
| PKN2 | RALB | 0 | 0 | 0 | 0 | 0.048 | 0.175 | 0 | 0.067 | 0.203 |
| PKN2 | PMAIP1 | 0 | 0 | 0 | 0 | 0 | 0 | 0 | 0.156 | 0.156 |
| PKN2 | THEM4 | 0 | 0 | 0 | 0 | 0 | 0 | 0 | 0.306 | 0.306 |
| PMAIP1 | TRAF4 | 0 | 0 | 0 | 0 | 0.065 | 0 | 0 | 0.147 | 0.168 |
| PMAIP1 | TP53 | 0 | 0 | 0 | 0 | 0 | 0.175 | 0.9 | 0.814 | 0.983 |
| PMAIP1 | TNFRSF10C | 0 | 0 | 0 | 0 | 0 | 0 | 0 | 0.263 | 0.263 |
| PMAIP1 | VEGFA | 0 | 0 | 0 | 0 | 0 | 0 | 0 | 0.369 | 0.369 |
| RALB | TP53 | 0 | 0 | 0 | 0 | 0 | 0 | 0 | 0.223 | 0.223 |
| TNFRSF10C | TP53 | 0 | 0 | 0 | 0 | 0.062 | 0 | 0.9 | 0.451 | 0.944 |
| TP53 | TRAF4 | 0 | 0 | 0 | 0 | 0.062 | 0 | 0 | 0.302 | 0.317 |
| TP53 | WNT7A | 0 | 0 | 0 | 0 | 0 | 0 | 0 | 0.324 | 0.323 |
| TP53 | VEGFA | 0 | 0 | 0 | 0 | 0 | 0.161 | 0 | 0.81 | 0.834 |
| VEGFA | WNT7A | 0 | 0 | 0 | 0 | 0 | 0 | 0 | 0.391 | 0.39 |

**Table S2. GO enrichment analysis**

| **Category** | **Term** | **Description** | **LogP** | **Log(q-value)** | **Enrichment factor** |
| --- | --- | --- | --- | --- | --- |
| GO Biological Processes | GO:0010942 | positive regulation of cell death | -15.86920651 | -11.68112214 | 0.025764895 |
| GO Biological Processes | GO:0043065 | positive regulation of apoptotic process | -15.36099139 | -11.47393702 | 0.027985075 |
| GO Biological Processes | GO:0043068 | positive regulation of programmed cell death | -15.1614461 | -11.45048298 | 0.027124774 |
| GO Biological Processes | GO:0032872 | regulation of stress-activated MAPK cascade | -12.84074616 | -9.284343228 | 0.051813472 |
| GO Biological Processes | GO:0070302 | regulation of stress-activated protein kinase signaling cascade | -12.7734576 | -9.284343228 | 0.051020408 |
| GO Biological Processes | GO:0080135 | regulation of cellular response to stress | -12.10287228 | -8.692939156 | 0.019283747 |
| GO Biological Processes | GO:0043408 | regulation of MAPK cascade | -11.05170043 | -7.708714093 | 0.018705036 |
| GO Biological Processes | GO:0032874 | positive regulation of stress-activated MAPK cascade | -10.94188147 | -7.687617116 | 0.062015504 |
| GO Biological Processes | GO:0070304 | positive regulation of stress-activated protein kinase signaling cascade | -10.88782962 | -7.687617116 | 0.061068702 |
| GO Biological Processes | GO:0001934 | positive regulation of protein phosphorylation | -10.87570149 | -7.687617116 | 0.01810585 |
| GO Cellular Components | GO:0048786 | presynaptic active zone | -5.239558279 | -1.964246925 | 0.048780488 |
| GO Cellular Components | GO:0031904 | endosome lumen | -4.62433928 | -1.650057921 | 0.076923077 |
| GO Cellular Components | GO:0005911 | cell-cell junction | -4.091105952 | -1.484532097 | 0.011764706 |
| GO Cellular Components | GO:0005667 | transcription regulator complex | -4.067848414 | -1.484532097 | 0.011650485 |
| GO Cellular Components | GO:0098802 | plasma membrane signaling receptor complex | -4.052993755 | -1.484532097 | 0.015673981 |
| GO Cellular Components | GO:0043235 | receptor complex | -3.981692201 | -1.484532097 | 0.011235955 |
| GO Cellular Components | GO:0017053 | transcription repressor complex | -3.720798183 | -1.335707714 | 0.038461538 |
| GO Cellular Components | GO:0030027 | lamellipodium | -3.707929082 | -1.335707714 | 0.01980198 |
| GO Cellular Components | GO:0030424 | axon | -3.58194426 | -1.260875415 | 0.009478673 |
| GO Cellular Components | GO:0031252 | cell leading edge | -3.488941784 | -1.213630429 | 0.011848341 |
| GO Molecular Functions | GO:0019900 | kinase binding | -5.9457862 | -2.250041925 | 0.011688312 |
| GO Molecular Functions | GO:0031625 | ubiquitin protein ligase binding | -5.378418586 | -2.007687237 | 0.019933555 |
| GO Molecular Functions | GO:0044389 | ubiquitin-like protein ligase binding | -5.226310258 | -2.007687237 | 0.01875 |
| GO Molecular Functions | GO:0070851 | growth factor receptor binding | -4.359541839 | -1.41995302 | 0.02919708 |
| GO Molecular Functions | GO:0019904 | protein domain specific binding | -4.330073965 | -1.41995302 | 0.010248902 |
| GO Molecular Functions | GO:0019901 | protein kinase binding | -4.318062652 | -1.41995302 | 0.010204082 |
| GO Molecular Functions | GO:0042803 | protein homodimerization activity | -4.270599256 | -1.41995302 | 0.010028653 |
| GO Molecular Functions | GO:0030546 | signaling receptor activator activity | -4.114616486 | -1.321962197 | 0.011881188 |
| GO Molecular Functions | GO:0046982 | protein heterodimerization activity | -3.905447327 | -1.221763961 | 0.014577259 |
| GO Molecular Functions | GO:0030545 | signaling receptor regulator activity | -3.894597551 | -1.221763961 | 0.010830325 |

**Table S3. KEGG enrichment analysis**

| **Category** | **Term** | **Description** | **LogP** | **Log(q-value)** | **Enrichment factor** |
| --- | --- | --- | --- | --- | --- |
| KEGG Pathway | hsa05200 | Pathways in cancer | -21.74354932 | -19.19700665 | 0.035781544 |
| KEGG Pathway | hsa04010 | MAPK signaling pathway | -19.25260278 | -17.00709011 | 0.051020408 |
| KEGG Pathway | hsa04210 | Apoptosis | -14.38042482 | -12.31100342 | 0.073529412 |
| KEGG Pathway | hsa05169 | Epstein-Barr virus infection | -10.98155044 | -9.037067767 | 0.044554455 |
| KEGG Pathway | hsa05417 | Lipid and atherosclerosis | -10.73870649 | -8.891133826 | 0.041860465 |
| KEGG Pathway | hsa05224 | Breast cancer | -10.48416159 | -8.715770182 | 0.054421769 |
| KEGG Pathway | hsa04151 | PI3K-Akt signaling pathway | -10.22899008 | -8.527545461 | 0.028248588 |
| KEGG Pathway | hsa05161 | Hepatitis B | -10.14539941 | -8.501946736 | 0.049382716 |
| KEGG Pathway | hsa05167 | Kaposi sarcoma-associated herpesvirus infection | -9.520739008 | -7.928438854 | 0.041237113 |
| KEGG Pathway | hsa05166 | Human T-cell leukemia virus 1 infection | -9.056813145 | -7.510270481 | 0.036036036 |

**Table S4. Top 10 targets calculated by betweenness method**

| **Rank** | **Name** | **Score** |
| --- | --- | --- |
| 1 | TP53 | 304.3435831 |
| 2 | FOS | 127.9677914 |
| 3 | IL6 | 115.7273334 |
| 4 | FOXO3 | 94.51479523 |
| 5 | VEGFA | 54.87574785 |
| 6 | DUSP1 | 47.21750942 |
| 7 | MAP2K4 | 39.59972197 |
| 8 | DDIT3 | 33.33091076 |
| 9 | PMAIP1 | 32.84009184 |
| 10 | CD40 | 30.10703741 |

**Table S5. Top 10 targets calculated by bottleneck method**

| **Rank** | **Name** | **Score** |
| --- | --- | --- |
| 1 | TP53 | 11 |
| 2 | FOXO3 | 6 |
| 3 | VEGFA | 5 |
| 4 | IL6 | 4 |
| 5 | FOS | 3 |
| 5 | PMAIP1 | 3 |
| 5 | CD40 | 3 |
| 8 | CTBP2 | 2 |
| 8 | MAP2K4 | 2 |
| 8 | GADD45A | 2 |

**Table S6. Top 10 targets calculated by closeness method**

| **Rank** | **Name** | **Score** |
| --- | --- | --- |
| 1 | TP53 | 40.5 |
| 2 | FOS | 38 |
| 3 | IL6 | 36.5 |
| 4 | FOXO3 | 35 |
| 4 | VEGFA | 35 |
| 6 | DDIT3 | 33 |
| 6 | DUSP1 | 33 |
| 8 | CD40 | 32.5 |
| 9 | FAS | 32 |
| 9 | MAP2K4 | 32 |

**Table S7. Top 10 targets calculated by degree method**

| **Rank** | **Name** | **Score** |
| --- | --- | --- |
| 1 | TP53 | 39 |
| 2 | FOS | 34 |
| 3 | IL6 | 31 |
| 4 | FOXO3 | 28 |
| 4 | VEGFA | 28 |
| 6 | DDIT3 | 24 |
| 6 | DUSP1 | 24 |
| 8 | CD40 | 23 |
| 9 | FAS | 22 |
| 9 | MAP2K4 | 22 |

**Table S8. Top 10 targets calculated by EPC method**

| **Rank** | **Name** | **Score** |
| --- | --- | --- |
| 1 | TP53 | 16.609 |
| 2 | FOS | 16.197 |
| 3 | FOXO3 | 15.568 |
| 4 | IL6 | 15.42 |
| 5 | VEGFA | 15.157 |
| 6 | DDIT3 | 15.009 |
| 7 | CD40 | 14.735 |
| 8 | DUSP1 | 14.404 |
| 9 | HSPA1A | 14.014 |
| 10 | MAP2K4 | 13.934 |

**Table S9. Top 10 targets calculated by MCC method**

| **Rank** | **Name** | **Score** |
| --- | --- | --- |
| 1 | TP53 | 6.13E+08 |
| 2 | IL6 | 6.13E+08 |
| 3 | FOS | 6.13E+08 |
| 4 | FOXO3 | 6.13E+08 |
| 5 | FAS | 6.11E+08 |
| 6 | DDIT3 | 6.08E+08 |
| 7 | VEGFA | 6.08E+08 |
| 8 | HSPA1A | 6.08E+08 |
| 9 | CD40 | 6.04E+08 |
| 10 | DUSP1 | 5.64E+08 |

**Table S10. Top 10 targets calculated by MNC method**

| **Rank** | **Name** | **Score** |
| --- | --- | --- |
| 1 | TP53 | 38 |
| 2 | FOS | 34 |
| 3 | IL6 | 31 |
| 4 | FOXO3 | 28 |
| 4 | VEGFA | 28 |
| 6 | DDIT3 | 24 |
| 6 | DUSP1 | 24 |
| 8 | CD40 | 23 |
| 9 | FAS | 22 |
| 9 | MAP2K4 | 22 |

**Table S11. Top 10 targets calculated by stress method**

| **Rank** | **Name** | **Score** |
| --- | --- | --- |
| 1 | TP53 | 1038 |
| 2 | FOS | 684 |
| 3 | IL6 | 598 |
| 4 | FOXO3 | 504 |
| 5 | VEGFA | 378 |
| 6 | DUSP1 | 266 |
| 7 | DDIT3 | 226 |
| 8 | MAP2K4 | 224 |
| 9 | CD40 | 202 |
| 10 | PMAIP1 | 182 |
